# Recurrent Embolic Strokes in a Patient with Anorectal Mucosal Melanoma: A Novel Presentation with Diagnostic and Therapeutic Challenge

**DOI:** 10.51894/001c.144501

**Published:** 2025-09-30

**Authors:** Anirudhan Ramaseshan, Ali Hussain

**Affiliations:** 1 College of Osteopathic Medicine Michigan State University; 2 Henry Ford Macomb

## 113

### ABSTRACT

Anorectal mucosal melanoma is a rare and aggressive malignancy often associated with poor prognosis. Its systemic implications, including a potential predisposition to thromboembolic events, remain poorly understood. This case report highlights the first documented occurrence of recurrent embolic strokes in a patient with anorectal mucosal melanoma. It is also the first documented case of stroke in mucosal melanoma without cardiogenic cause or brain metastasis. The diagnostic challenges and therapeutic considerations are discussed, underscoring the need for further research into the interplay between hypercoagulability and malignancy.

### INTRODUCTION

Mucosal melanoma is a rare and aggressive form of melanoma, accounting for less than 1% of all melanoma cases. Commonly arising in the head and neck, vulvovaginal, or anorectal regions, it is typically diagnosed at advanced stages and is associated with poor prognosis.[4] Unlike cutaneous melanoma, mucosal melanoma lacks a clear link to ultraviolet exposure and demonstrates distinct biological characteristics, such as a lower frequency of BRAF mutations and unique metastatic patterns. Local excision remains the primary treatment, but recurrence and metastasis are common, necessitating systemic therapies (i.e. immunotherapy and targeted agents such as BRAF and KIT inhibitors). [5]

While hypercoagulability is a recognized paraneoplastic phenomenon in melanoma, its manifestation as recurrent embolic strokes in mucosal melanoma without cardiogenic cause or brain metastasis has not been reported. [1,2] This case highlights the diagnostic and therapeutic challenges of managing thromboembolic events in this context and underscores the importance of multidisciplinary collaboration.

### CASE PRESENTATION

A 64-year-old male with a history of anorectal mucosal melanoma presented in November 2024 with acute onset of dysarthria and right facial droop. He had a prior history of a thrombotic stroke in June 2024 involving the right middle cerebral artery (MCA), managed with thrombectomy. Post-thrombectomy imaging revealed chronic high-grade stenosis of the right MCA, and the patient was maintained on dual antiplatelet therapy with aspirin and clopidogrel. Additional medical history included gastroesophageal reflux disease (GERD), generalized anxiety disorder (GAD), hypertension, and dyslipidemia.

